# Increased risk of postsurgical macular edema in high stage idiopathic epiretinal membranes

**DOI:** 10.1186/s40662-021-00252-4

**Published:** 2021-08-04

**Authors:** Lorenzo Iuliano, Gloria Cisa di Gresy, Giovanni Fogliato, Eleonora Corbelli, Francesco Bandello, Marco Codenotti

**Affiliations:** Department of Ophthalmology, Vita-Salute University, San Raffaele Scientific Institute, Via Olgettina 60, 20132 Milan, Italy

**Keywords:** Epiretinal membrane, Macular edema, Macular hole, Vitrectomy, Postsurgical macular edema

## Abstract

**Purpose:**

To assess the rate of occurrence and the risk factors of postsurgical macular edema (PSME) in eyes with idiopathic epiretinal membrane (iERM) or full-thickness macular hole (FTMH).

**Methods:**

Retrospective longitudinal analysis of all subjects scheduled for vitrectomy with or without combined cataract surgery over a 6-month period. Electronic medical charts and imaging data were analyzed preoperatively and at 1, 3 and 6 months after surgery.

**Results:**

From 101 patients diagnosed with iERM or FTMH, 71 patients were eligible for the study. Forty-nine eyes with iERM (69.0%) and 22 eyes with FTMH (31.0%) underwent vitrectomy either isolated (31.0%) or combined with cataract extraction (69.0%). The overall rate of PSME was 26.7%, without differences between the two groups (*P* = 0.9479). Combined cataract extraction did not affect the overall occurrence of PSME rate in both groups (*P* = 0.9255 in FTMH and *P* = 0.8658 in iERM). If grouped by stage, eyes with stage 4 iERM though disclosed an increased rate of PSME (57.1%) compared to lower (1 to 3) stages (14.3%, *P* = 0.0021), particularly when combined with cataract surgery (71.4% vs. 15.4% in stages ≤ 3, *P* = 0.0021). The PSME odds ratio for a stage 4 iERM is 8 (95% CI: 1.933–33.1; *P* = 0.0041) compared to stages 3 and below.

**Conclusions:**

PSME remains a clinically relevant and frequent event after surgery for iERM and FTMH. Patients with stage 4 iERM have an 8-fold higher likelihood of developing PSME in a 6-month postsurgical period compared to iERM in 1–3 stages, especially when combined with cataract extraction.

## Background

Postsurgical macular edema (PSME) may complicate several intraocular procedures, notably surgery for glaucoma, corneal transplant and, most commonly, cataract extraction [1–5]. It has been recognized as the most common cause of visual deterioration after uncomplicated cataract surgery, with rates ranging from 0.1 to 3.8%, and it is specifically known as Irvine-Gass syndrome. In relation to cataract surgery, well-established risk factors include intraoperative complications such as posterior capsule rupture and preoperative factors such as diabetes mellitus, uveitis, retinal vein occlusion and epiretinal membrane (ERM) [1].

The pathogenesis is ascribed to the breakdown of the blood-aqueous barrier due to an amplified inflammatory reaction and release of cytokines [1, 5–10].

Postsurgical macular edema has also been identified following successful vitreoretinal surgery, ranging from macular surgery [11–13] to ab externo [14, 15] and ab interno [16] repair of retinal detachment. This unpleasant condition after retinal surgery has been reported to occur with different frequencies, from 10 to 47% [11–13]. Cataract surgery, which can be addressed to be a confounding factor in the case of combined phacovitrectomy, has been demonstrated not to influence the rate of PSME in macular surgeries [12, 17–20].

This study aims to estimate the rate of occurrence of PSME after vitrectomy for idiopathic epiretinal membrane (iERM) and full-thickness macular hole (FTMH), further contributing to evaluate the possible risk factors associated with its manifestation.

## Materials and methods

### Ethics

All the procedures performed involving human participants were in accordance with the ethical standards of the Institutional Review Board of the San Raffaele Scientific Institute and with the 1964 Declaration of Helsinki and its later amendments. All patients, at the time of hospital admission, signed a general informed consent form that was specifically designed and approved by the Institutional Review Board of the San Raffaele Scientific Institute solely for observational retrospective studies. Patients signing this form consent to use their anonymized clinical data for the purpose of designing retrospective investigations.

### Study participants

All subjects were recruited at the Vitreoretinal Surgery Service of the Ophthalmology Department, San Raffaele Scientific Institute. Inclusion criteria were: age 18 years or older, presence of iERM or idiopathic FTMH and availability of least 6 months of follow-up in the institutional electronic medical charts. Patients were diagnosed by biomicroscopy and spectral-domain optical coherence tomography (SD-OCT) (Spectralis-OCT; Heidelberg Engineering, Heidelberg, Germany). Exclusion criteria were: iERM or FTMH secondary to any ocular disease (e.g., diabetic retinopathy, uveitis, retinal vein occlusion, retinal dystrophies, etc.), glaucoma, history of head or ocular trauma, previous anterior segment or posterior segment surgery (including retinal laser photocoagulation) within 12 months. Pseudophakic eyes with a previous history of macular edema after cataract surgery were excluded, as do eyes that developed postsurgical complications (such as retinal detachments, vascular occlusions, optic neuropathies, etc.). Subjects whose imaging acquisition was jeopardized by any optical media opacity (cornea or lens) were not enrolled. Individuals with diabetes without retinopathy were also excluded. In case both eyes were eligible, the first operated eye was included in the study.

### Study protocol

In this longitudinal retrospective study, we reviewed the electronic medical charts of all the institutional patients affected by iERM and FTMH operated from June 2018 to December 2018. All patients were visited and operated at the Vitreoretinal Surgery Service of San Raffaele Scientific Institute in Milan, a tertiary-referral ophthalmological center.

All institutional patients routinely undergo a preoperative ophthalmological evaluation, then standard postoperative controls at 1 week and at 1, 3 and 6 months. SD-OCT is, per protocol, always performed in addition to the clinical evaluation on every preoperative and postoperative control except at the 1-week mark. For this reason, this point of observation was excluded from the analysis.

### Investigated variables

Data gathered during preoperative examination included: general and ocular medical history, age, sex, distance best-corrected visual acuity (BCVA), metamorphopsia assessment (with Amsler grid test) and SD-OCT examination.

Distance BCVA is regularly measured in dedicated offices with continuously monitored luminance, using the Early Treatment in Diabetic Retinopathy Study (ETDRS) charts. The Snellen notation extracted from the electronic medical charts was converted into logMAR for ease of calculations [21].

SD-OCT images are performed in our center using the Spectralis OCT device (Heidelberg Engineering, Heidelberg, Germany). Macular raster scans are routinely acquired after pupil dilation to image the macular region using the follow up function. For the research purposes of this study, the central foveal thickness (CFT) was defined as the mean thickness within the central 1000 μm diameter area (the central circle on the ETDRS map).

**Fig. 1:**
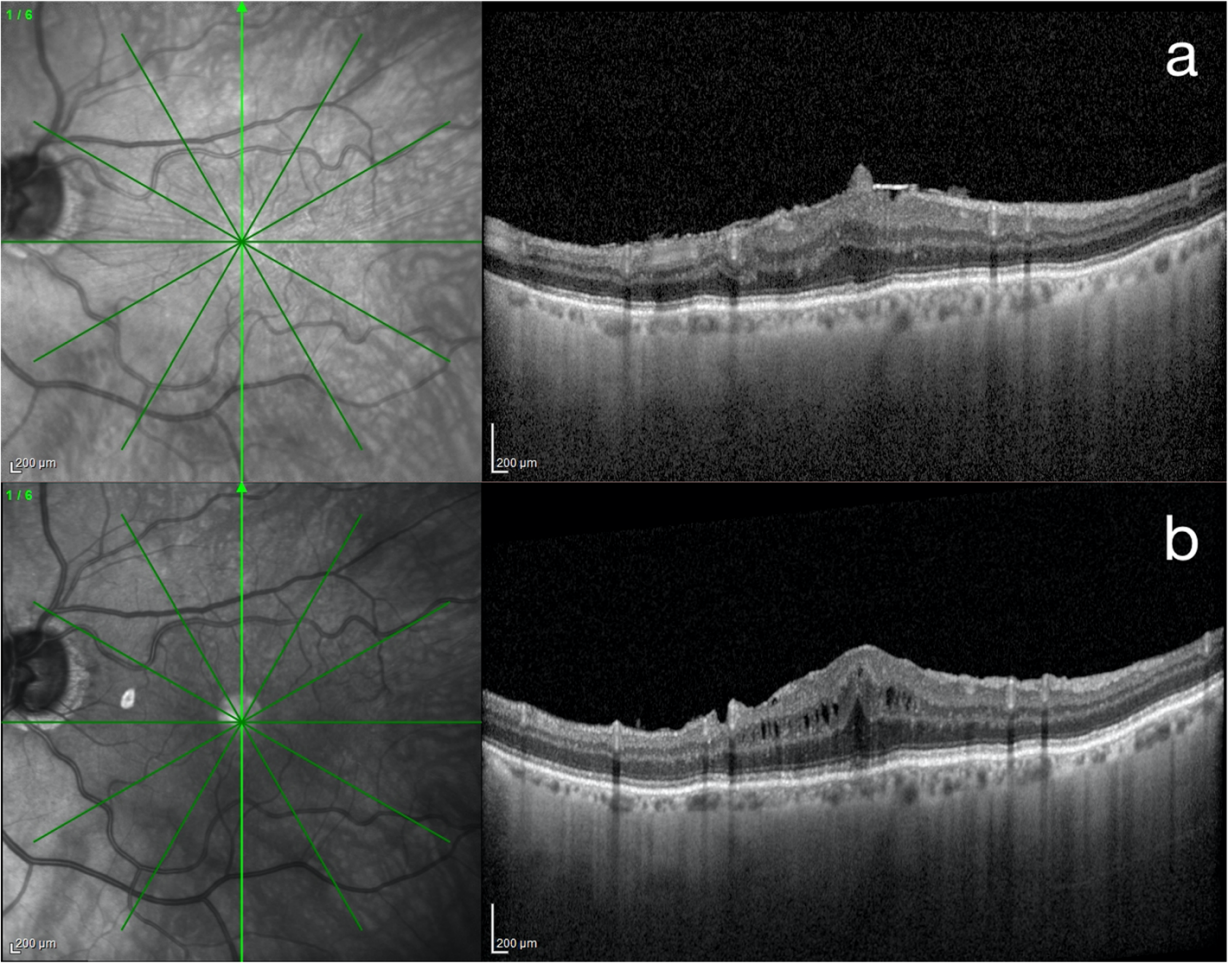
Postsurgical macular edema after surgery for stage 3 idiopathic epiretinal membrane. Preoperative (a) 30° combined infrared reflectance and structural optical coherence tomography vertical B-scan passing through the fovea of a case of stage 3 idiopathic epiretinal membrane. Characteristic features are the ectopic inner foveal layer and the absence of the foveal depression. One month after surgery (b) cystoid macular edema is evident, with increased central foveal thickness

The presence of PSME was assigned only in those cases that showed, during the observation period after surgery: 1) a new onset of macular hyporeflective cystoid intraretinal spaces at SD-OCT (Fig. 1) that were not present before vitrectomy; 2) definite worsening in size and number of pre-existing cystoid spaces with an increase in CFT of 10% or more over baseline.

Idiopathic ERMs were staged using the staging system by Govetto et al. [22]. The classification considers 4 stages: stage 1 is defined as the presence of a mild iERM with negligible morphologic or anatomic disruption and preserved foveal depression; stage 2, in case of presence of an iERM associated with more progressive retinal distortion and absence of foveal depression; stage 3 is defined as the presence of an iERM with continuous ectopic inner foveal layers (EIFLs) anomalously crossing the central foveal area with all retinal layers clearly identifiable on OCT; stage 4 are iERMs complicated by significant retinal thickening and remarkable anatomic disruption of the macula. Continuous EIFL is defined on OCT as the presence of a continuous hyporeflective or hyperreflective band, extending from the inner nuclear layer and inner plexiform layer across the foveal region and visible in all OCT scans centered in the fovea.

FTMHs were classified according to the International Vitreomacular Traction Study Group [23], that defines holes ≤ 250 μm as small, > 250 and ≤ 400 μm as intermediate, > 400 μm as large.

Combined cataract and vitreoretinal surgery were performed if clinically significant lens opacifications were present, according to the surgeon’s decision.

### Surgical procedure

Twenty-five-gauge transconjunctival three-port pars plana sutureless vitrectomy with ERM and internal limiting membrane (ILM) peeling was successfully performed in all cases by a single experienced vitreoretinal surgeon (MC). ILM peeling was obtained with the pinch technique, grasping the ILM nearby the macula with macular forceps and providing a round maculorrhexis. Membranes (ERM and ILM) were peeled in all patients with the conjugated dye TWIN, consisting of 0.18% trypan blue + 0.03% blulife (AL.CHI.MI.A., Italy). Fluid-air and (if needed) air-gas exchanges were performed in appropriate cases. Concurrent standard phacoemulsification was done in all cases placing a single-piece intraocular lens (Artis PLE) in the capsular bag.

### Treatment

Standard postsurgical therapy was: topical association of netilmicin 3 mg/ml and dexamethasone 1 mg/ml drops 4 times a day for 4 weeks, and tropicamide 10 mg/ml drops twice a day for 1 week.

The treatment regimen for the management of PSME in our center is: dexamethasone 2 mg/ml drops 4 times a day plus bromfenac 0.9 mg/ml drops twice a day for 4 weeks. According to the clinician’s judgement of the therapeutic effects, treatment can be halted, tapered or switched to second line agents that includes intravitreal steroids (triamcionolone acetonide 80 mg/ml or dexamethasone implant 700 μg).

### Statistical analysis

Statistical analyses were performed using GraphPad Prism version 5.00 for Mac (GraphPad Software, San Diego, California, USA) and Past 4.0 (Palaeontologica Electronica). Distribution normality was tested with the Shapiro-Wilk test. The Chi-squared test was used to compare non-continuous variables. The Friedman test and the Wilcoxon test were applied to compare the quantitative measures throughout the follow-up within the same group. The Mann-Whitney test was used to compare the outcomes between the two investigational groups. In all analyses, *P*-values < 0.05 were considered significant.

Considering the explorative nature of the research, we studied a certain convenience sample of patients, based on a 6-month subject availability. The alternative hypotheses H1 were that cataract surgery or the iERM stage could generate a difference in PSME rate. The study sample size was calculated with a power of 0.8 and alpha value of 0.05.

## Results

### Population

One hundred and one eyes of 101 consecutive patients diagnosed with either iERM or FTMH and with 6 months of follow-up were enrolled. Of these eyes, 30 were excluded because of the presence of one or more exclusion criteria, of which 5 were due to postsurgical complications (3 rhegmatogenous retinal detachments, 2 central retinal vein occlusions). At the end of the selection process, 71 eyes of 71 patients were enrolled, of which 39 were females (54.9%) and 32 males (45.1%), with an average age of 71 ± 7 years (range 51–91 years) (Table 1). Continuous variables (BCVA, CFT) turned out to be normally distributed.

**Table 1:** Baseline clinical features of the two groups

	Full-thickness macular hole	Idiopathic epiretinal membrane	*P*
Eyes	22	49	–
Sex (Femal:Male)	12:10	26:23	0.9076
Age (years)	69 ± 5	72 ± 4	0.3035
BCVA (logMAR)	0.77 ± 0.27 (~ 20/125)	0.42 ± 0.22 (~ 20/50)	< 0.0001
CFT (μm)	339 ± 28	446 ± 38	< 0.0001

*BCVA* best-corrected visual acuity; *logMAR* logarithm of minimum angle of resolution; *CFT* central foveal thickness

Of the 71 enrolled eyes, 49 (69.0%) were diagnosed with iERM and 22 (31.0%) with FTMH.

Among the eyes with iERM, 2 eyes (4.0%) were stage 1; 9 (18.4%) were stage 2; 24 (49.0%) were stage 3 and 14 (28.5%) were stage 4.

As far as eyes diagnosed with FTMH, average macular hole diameter was 328 ± 75 μm. Three eyes (13.6%) had a large macular hole (MH), 17 (77.3%) had an intermediate MH and 2 (9%) were small MH. Closure rate was 100%.

### Surgery

Twenty eyes with FTMH (90.9%) received endotamponade with 10% SF_6_, while the 2 small FTMHs were closed under air.

In 7 eyes with iERM (14.3%), fluid-air exchange was done at the end of surgery due to the intraoperative finding of peripheral holes (6 eyes) or iatrogenic retinal tear (1 eye) without detachment. Holes were treated with endolaser whereas the tear with cryopexy.

Combined phacovitrectomy was performed in 49 of the 71 cases (69.0%), while the remaining 22 eyes (31.0%) underwent an isolated vitreoretinal procedure. Out of these 22 eyes, 13 (59.0%) previously underwent an uncomplicated cataract extraction 32.4 ± 10 months (range 19–49 months) earlier.

Combined phacovitrectomy was done in similar proportions between iERM (34 out of 49, 69.4%) and FTMH (15 out of 22, 68.2%; *P* = 0.9190) groups.

### Visual acuity

The distance BCVA significantly improved throughout the follow-up in both iERM and FTMH groups (Table 2).

**Table 2:** Average best-corrected visual acuity and central foveal thickness changes throughout the follow-up in the two investigational groups

		Baseline	1 month	3 months	6 months	*p*
BCVA (logMAR)	FTMH	0.93	0.53	0.37	0.36	0.0006
	iERM	0.42	0.32	0.26	0.26	0.0018
CFT (μm)	FTMH	339.33	300.67	312.50	292.33	0.2423
	iERM	444.10	423.60	395.10	333.20	0.0013

*BCVA* best-corrected visual acuity; *logMAR* logarithm of minimum angle of resolution; *FTMH* full-thickness macular hole; *iERM* idiopathic epiretinal membrane; *CFT* central foveal thickness

Eyes with iERM disclosed at baseline higher BCVA (~ 20/50) on average than patients with FTMH (~ 20/125), however no statistically significant difference was noted in the trends between the two groups at each follow-up visit (*P* > 0.05, not significant for all).

Within the iERM group, the BCVA of eyes affected by PSME was found to be, at the last follow-up (6 months), averagely deteriorated (~ 20/100) and statistically similar (*P* = 0.2070) to presurgical values (~ 20/80). Eyes that did not develop PSME improved their vision, from ~ 20/50 before surgery to ~ 20/25 at the last follow-up visit (Table 3).

**Table 3:** Change of distance best-corrected visual acuity from baseline (before surgery) to the 6-month visit in idiopathic epiretinal membranes and full-thickness macular holes grouped by the occurrence of postsurgical macular edema

		Baseline	6 months	*P*
iERM	No PSME	0.35 ± 0.17 (~ 20/50)	0.13 ± 0.10 (~ 20/25)	0.0051
	PSME	0.58 ± 0.06 (~ 20/80)	0.67 ± 0.09 (~ 20/100)	0.2070
FMTH	No PSME	0.81 ± 0.24 (~ 20/125)	0.38 ± 0.30 (~ 20/40)	0.0006
	PSME	0.75 ± 0.35 (~ 20/125)	0.95 ± 0.32 (~ 20/160)	0.0625

The reported values are mean ± standard deviation units of the minimum angle of resolution (logMAR)

*iERM* idiopathic epiretinal membrane; *PSME* postsurgical macular edema; *FMTH* full-thickness macular hole

Similar trends were found within the FTMH group, as the BCVA of eyes that developed PSME did not improve after surgery (from ~ 20/125 to ~ 20/160; *P* = 0.0625), but improved in eyes without PSME (Table 3).

### Postsurgical macular edema

CFT significantly reduced after surgery in the iERM groups, whereas remained substantially unchanged in the FTMH group (Table 2). Patients with iERM disclosed at baseline thicker fovea on average than patients with FTMH.

**Fig. 2:**
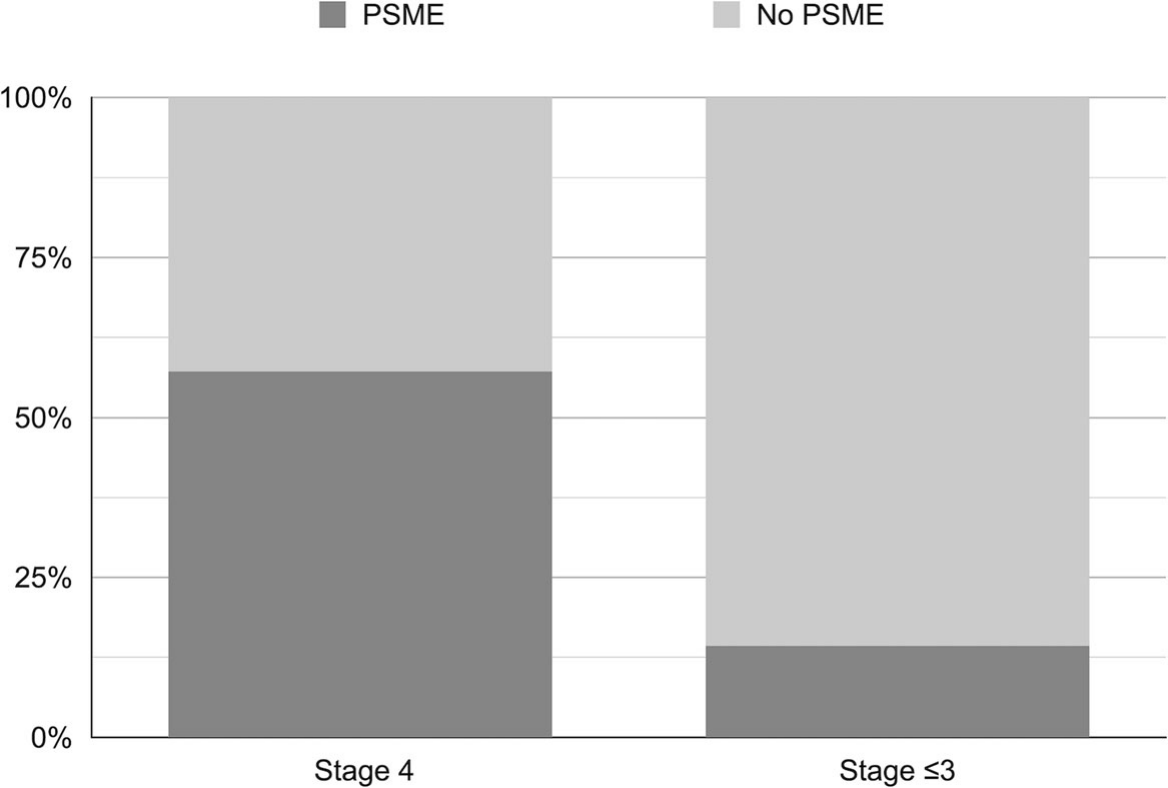
Rate of postsurgical macular edema according to the epiretinal membrane stage. Stage 4 idiopathic epiretinal membranes disclose higher rate of postsurgical macular edema compared to overall rate of less-than-4 stages

The global frequency of PSME (according to the definition criteria) was 26.7%. This rate was on average similar between the FTMH group (6 out of 22, 27.3%) and the iERM group (13 out of 49, 26.5%) (*P* = 0.9479). Within the iERM group, PSME occurrence turned out to be significantly higher in stage 4 iERMs (8 out of 14 eyes, 57.1%) compared to stages lower than 4 (5 out of 35, 14.3%) (*P* = 0.0021, Fig. 2). The PSME odds ratio for a stage 4 iERM is 8 (95% CI: 1.933–33.1; *P* = 0.0041) compared with stages 3 and below.

**Fig. 3:**
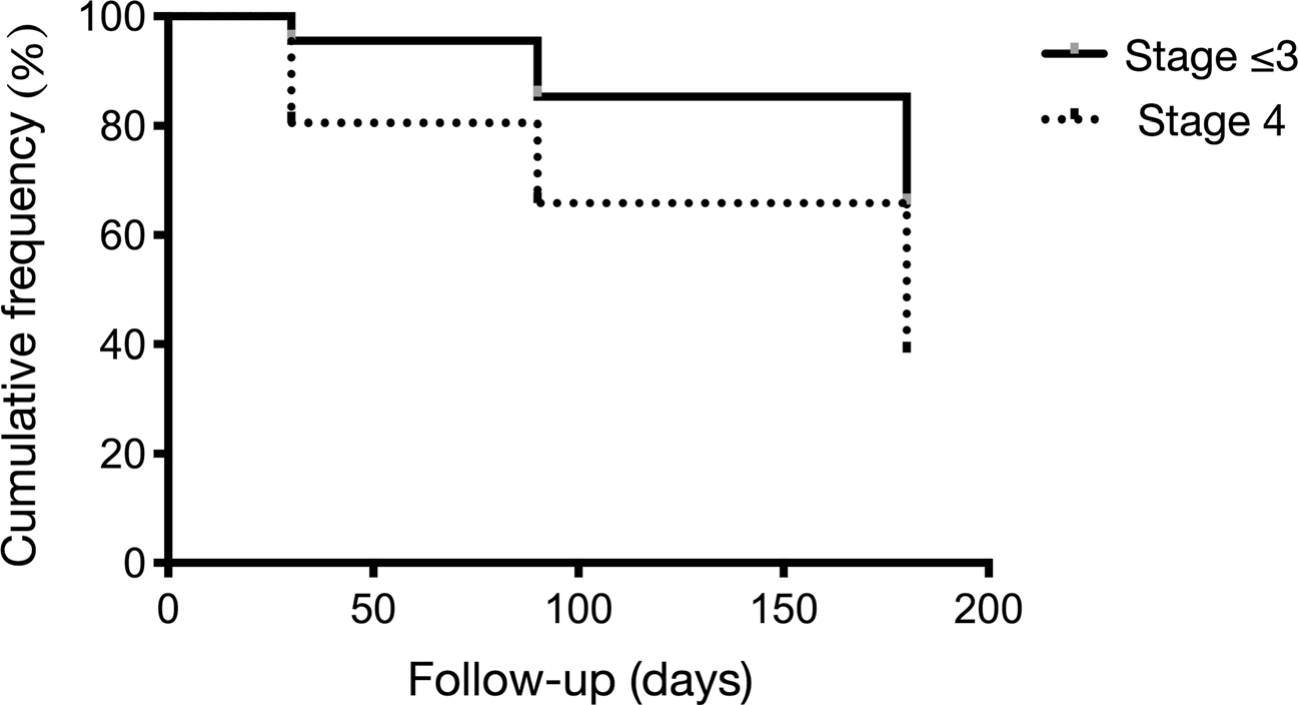
Cumulative frequency of postsurgical macular edema according to the epiretinal membrane stage. The cumulative frequency of postsurgical macular edema throughout the follow-up significantly reduced, with a reduction rate greater for stage 4 idiopathic epiretinal membranes with respect to stages 1–3

No subjects in stage 1 developed edema after surgery. The PSME cumulative frequency throughout the follow-up significantly reduced, with a higher reduction rate for stage 4 iERMs with respect to the other stages (Fig. 3).

### Combined procedures

Adjunct phacoemulsification to pars plana vitrectomy did not influence the overall PSME rate in the FTMH group. The proportion of edema in combined surgery was 26.7% (4 out of 15) and 28.6% (2 out of 7) in the isolated vitreoretinal procedure (*P* = 0.9255). Analogously, despite the rate of PSME in the iERM group being slightly superior in combined surgeries (27.3%, 9 out of 33) compared with isolated vitreoretinal procedures (25.0%, 4 out of 16), this did not achieve statistical significance (*P* = 0.8658).

Additional sub-analysis disclosed that stage 4 iERMs were most prone to develop PSME when subject to combined phacovitrectomy surgery. Combined surgery was performed in 50.0% of eyes with stage 4 iERMs (7 out of 14 eyes) and in 77.1% of eyes with stage < 4 iERMs (27 out of 35 eyes). Within the 9 cases with iERM that developed PSME in the phacovitrectomy group (as above-mentioned), 71.4% were in stage 4 (5 out of the 7 eyes with a stage 4 iERM) and 14.8% were in stage < 4 (4 out of the 27 eyes with a stage < 4 iERM), being therefore the rate of PSME significantly greater in high stage iERMs (*P* = 0.0031).

### Treatment

All the 19 eyes that developed PSME received initial topical therapy according to the reported regimen. Twelve eyes were treated for 4 weeks, but in 4 eyes, drops were tapered over a longer period (up to 10 weeks). Two eyes received intravitreal triamcinolone and 1 eye was scheduled for dexamethasone implant.

## Discussion

Although the introduction of phacoemulsification technique has led to a significant decrease in frequency, PSME remains a therapeutic challenge for clinicians, especially when persisting for several months. The overall incidence of clinical PSME following cataract surgery is reported as 0.1 to 2.35% [1, 5, 24]. Angiographic findings are much more frequent, although variable, reaching 70% in some studies [25].

The etiology of PSME is not completely understood [10]. Hruby originally hypothesized some possible microenvironmental retinal changes producing the fluid accumulation: vitreous traction on the macula, vitreous incarceration in the wound, vitreous uveal traction, inflammation, light damage [1, 26–28]. Specifically, inflammation and increased levels of prostaglandins released by anterior segment structures are considered to play the most relevant role [5, 10, 28, 29]. These mediators can freely diffuse in the posterior segment and cause extravasation of fluid from the retinal vessels, which collect in cystic spaces within the retinal layers. The crucial role of inflammation can be additionally inferred by the demonstrated beneficial role of anti-inflammatory postoperative therapy in preventing macular edema [29–31].

Postsurgical macular edema can also occur after pars plana vitrectomy. Despite a common awareness of this condition, the clinical information available throughout literature as far as its incidence, effects on visual recovery and management are less systematic than “conventional” post-cataract Irvine-Gass syndrome [11–13].

In our study we found, by means of SD-OCT, an overall rate of PSME of 26.7%, similarly distributed in both iERMs and FTMHs.

With regards to iERM, this frequency may appear overestimated if compared to other studies (7.2% in the series of Leisser [11] and 10% in Frisina’s report [12]), and in line with Kim’s series (40%) [13]. Our sub-analysis highlighted the PSME rate turned out to be significantly greater in stage 4 (57.1%) compared to the other less-than-4 stages together (16.7%), which in turn seems to match with previously published data [11, 12]. We hypothesize our rate was hence influenced by the remarkably increased frequency of PSME of advanced iERM stages. The odds of developing macular edema after vitreoretinal surgery for a patient diagnosed with a stage 4 iERM is indeed 8-fold higher than for patients with membranes of lower stages.

A second remarkable consideration deals with the influence of cataract surgery on the PSME rate. Out of the 49 eyes that globally underwent combined phacovitrectomy, the proportion of these that developed PSME was on average similar between FTMH and iERM groups (~ 27%). Despite the iERM group undergoing combined surgery turned out to have a slightly superior proportion of eyes with PSME, this data did not reach statistical significance. Similar conclusions were also reported by Frisina and colleagues [12]. Therefore, combined cataract surgery and vitrectomy did not seem to influence the occurrence of macular edema.

The sub-analysis carried out on eyes with iERM undergoing combined surgery disclosed noteworthy conclusions. We found that within the overall 9 cases with iERM that developed PSME after phacovitrectomy, a higher proportion was in stage 4 (71.4% vs 14.8% in stage < 4), therefore the rate of PSME was significantly greater in the higher stage. Thus, patients with a stage 4 iERM are not only more prone to develop PSME in general, but are also more vulnerable in case of both surgeries simultaneously, rather than the single procedures separately.

A possible explanation for such susceptibility of stage 4 iERMs, compared to the other stages, can be searched in the pathogenesis of the disease itself. Stage 4 iERMs are complicated by significant retinal thickening and remarkable anatomic disruption of the macula, with continuous EIFLs that extend from the inner nuclear layer and inner plexiform layer of the retina, and cross the entire foveal area [32–34]. Of note, there is growing evidence that iERM formation, which previously was considered to be predominantly epiretinal, may have a significant intraretinal component [33, 35]. Therefore, we postulate that this state of retinal architectural disruption found in end-stage iERMs may predispose to further tissue changes when subject to mechanical stress, such as the peeling maneuvers. ILM peeling might indeed transmit further mechanical stress to the retinal glia, placing additional damage to the already suffering and markedly altered tissue.

Moreover, it has been postulated that PSME may derive from Müller cell dysfunction and not from blood-retina barrier disruption [36, 37]. This specific subtype of edema has been described as “microcystoid macular edema” (MME), which is not related with retinal vascular impairment, included in its determinants are retrograde trans-synaptic degeneration of inner retinal layers [38], inflammatory process [39] and vitreous traction [40]. The resultant effect is an impairment in fluid resorption in the macula, causing microvacuolar changes that do not represent true edema but rather fluid-filled empty spaces replacing degenerated cells [38, 41].

This evidence reinforces the hypothesis that this tissue susceptibility, in combination with mechanical stress and inflammation, may together play a key role in the persistency of PSME.

Eyes that developed PSME were found to lack any visual improvement by the end of the observation period, both in the iERM and FTMH groups. Those eyes that had an uneventful follow-up indeed improved their visual acuity by the last observation.

Some important limitations must be disclosed. First, owing to the fact that surgery is rarely proposed in lower iERM stages (1 and 2), the study was underpowered to detect relative differences when compared to higher stage holes (3 and 4). Second, given the retrospective study design, we could not review any fluorescein angiography data, that were never performed per protocol. Angiographic data might have allowed a comprehensive evaluation of the blood-retina barrier condition, further providing additional information regarding the neuro-degenerative or exudative etiology of edema. Electrophysiology, that was never performed, could have also improved some useful physiopathologic considerations.

Finally, FTMHs are often characterized by the preoperative presence of intraretinal cystoid spaces, whose pathophysiology seems not to involve any vascular component [42]. The distinction between exudative versus non-exudative pattern of these cystoid spaces can be clearly highlighted by multi-modal imaging, which we were not able to review as this was not part of routine procedure.

We eventually acknowledge to have relied on an OCT-based diagnosis of PSME, probably aggregating together mixed or distinct etiologies of edema. We believe this aspect, however, did not weaken the epidemiological value of the study, as it emphasizes the enhanced likelihood of PSME in advanced stage of iERMs, regardless the cause.

Strengths of our research were, on the other hand, the careful patient selection to achieve a homogeneous sample, as well as the relatively long follow-up period.

In conclusion, PSME remains an important and relatively frequent event after macular surgery, in both FTMH and iERM. It has been demonstrated that patients with better initial visual acuity achieve higher visual outcomes, but those with poorer preoperative vision show a greater functional change following ERM surgery [43]. Therefore, even eyes with advanced iERMs may retain a good prognosis and can be considered suitable for surgery. Appropriate prognostic factors associated with postoperative visual acuity, including the ellipsoid zone integrity [44–46], must undoubtedly be taken note of by clinicians. Owing to the increased risk of PSME in stage 4 iERMs, and to the related possible lack of visual improvement, we emphasize the importance of adding an accurate preoperative staging to the panel of prognostic factors to be explored.

It can also be argued that a separate procedure (cataract and vitrectomy) can achieve a better anatomical and functional outcome as well as whether surgery in earlier stages might be advisable. ERMs in advanced stages and the presence of EIFLs have indeed both demonstrated to negatively influence the functional outcomes after surgery [34, 47].

Despite the clinical considerations of our research, we believe our study enriches the clinician with simple but significantly important prognostic data for making informed decisions in the clinic.

## Conclusions

PSME is a clinically relevant and frequent event after surgery for both iERM and FTMH, with an overall rate of 26.7%. PSME occurrence turned out to be significantly higher in stage 4 iERMs (57.1%) compared to stages lower than 4 (14.3%). Stage 4 iERMs have an 8-fold higher likelihood of developing PSME in a 6-month postsurgical period compared to iERM in 1–3 stages, especially when combined with cataract extraction.

## Data Availability

The datasets used and/or analyzed during the current study are available from the corresponding author upon reasonable request.

## Abbreviations

BCVA: Best-corrected visual acuity
CFT: Central foveal thickness
EIFL: Ectopic inner foveal layer
FTMH: Full-thickness macular hole
iERM: Idiopathic epiretinal membrane
ILM: Internal limiting membrane
LogMAR: Logarithm of the minimal angle of resolution
MME: Microcystoid macular edema
PSME: Postsurgical macular edema
SD-OCT: Spectral-domain optical coherence tomography
